# *Nuttalliella namaqua* Bedford, 1931, a sole extant species of the genus *Nuttalliella* – a scoping review

**DOI:** 10.3389/fpara.2024.1401351

**Published:** 2024-06-27

**Authors:** Maphuti Betty Ledwaba, Dikeledi Petunia Malatji

**Affiliations:** Department of Agriculture and Animal Health, College of Agriculture and Environmental Sciences, University of South Africa, Roodepoort, South Africa

**Keywords:** Nuttalliellidae, *Nuttalliella*, *Nuttalliella namaqua*, life cycle and host preference, distribution

## Abstract

*Nuttalliella namaqua* Bedford, 1931 is the sole extant tick species that belongs to the genus and family *Nuttalliella* and Nuttalliellidae respectively. With the characteristics that are respectively distinctive to hard and soft ticks, it is regarded as the species closest to the ancestral lineage of ticks as well as the missing link between the Argasidae and Ixodidae families. In this review, literature search of the articles reporting on *N. namaqua* was done in Google Scholar and PubMed databases. After relevance and eligibility screening, 12 articles were deemed eligible and appraised. The results showed that *N. namaqua* was respectively distinct to limited regions of Africa such as Botswana, Namibia, Mozambique, South Africa and Tanzania. The review also indicated that *N. namaqua* was collected from murid rodents, African Savanna hare, scrub hare, elephant shrews, rock hyraxes, black backed jackal, lizards and off-host in locations that include under a stone, rock crevices, on a rock wall and respectively in the nests of an eagle and a lesser striped swallow. Irrespective of all the reports, natural hosts of the nymphs are still not clearly defined. Numerous phylogeny studies have reported Nuttalliellidae as the sister-lineage to Argasidae and Ixodidae tick families. Moreover, a recent report indicated that the similarities between Nuttalliellidae and the fossil families Deinocrotonidae and Legionaris award them to be merged into one family, preferably Nuttalliellidae Thus, further research on this family, will perhaps provide more knowledge about its unclear distribution, life cycle as well as the evolution of ticks in general.

## 1 Introduction

Ticks are significant ectoparasites of humans, domestic and wild animals (Jongejan and Uilenberg, 1994); and are distributed globally in different environmental conditions. They have a considerable importance in the medical and veterinary health as they harbor and transmit a wide range of pathogens causing diseases to their preferred hosts (Jongejan and Uilenberg, 1994; Dantas-Torres, 2010). Nuttalliellidae was an ancient monotypic tick family from the order Ixodida, which also comprises of Argasidae, Ixodidae as well as the three extinct fossil families Deinocrotonidae (Peñalver et al., 2017), Khimairidae (Chitimia-Dobler et al., 2022) and Legionaris (Chitimia-Dobler et al., 2024), which were described recently based on fossil tick specimens from Burmese amber deposits. At present, approximately 970 extant tick species have been described globally and Ixodidae is the largest and most prominent with ~750 species, followed by Argasidae with ~218 species, while Nuttalliellidae has a sole extant genus and species, *Nuttalliella* and *Nuttalliella namaqua* (Dantas-Torres and Otranto, 2022), as well as five extinct species, namely, *Nuttalliella gratae* sp. nov., *Nuttalliella odyssea* sp. nov., *Nuttalliella placaventrala* sp. nov., *Nuttalliella tropicasylvae* sp. nov. and *Nuttalliella tuberculata* sp. nov., that were described and placed within the genus *Nuttalliella* (Chitimia-Dobler et al., 2024). Deinocrotonidae had one species as well, however, an additional species has been described along with the fossil family Khimairidae (Chitimia-Dobler et al., 2022) and two more species described recently from Burmese fossil deposits as well (Chitimia-Dobler et al., 2024). Moreover, the above-mentioned authors also reported that the notable similarities between the genera *Nuttalliella*, *Deinocroton* and the newly described *Legionaris* justifies the addition of the latter two genera in the family Nuttalliellidae besides *Nuttalliella*. Even though ticks are ranked second to mosquitoes as vectors of the bacterial, protozoan and viral disease-causing pathogens of animals and humans (Jongejan and Uilenberg, 1994; Parola and Raoult, 2001), the potential of *N*. *namaqua* being a vector is still not established; and it has not been associated to any pathogen/disease since its description.

The acknowledged names (Nuttalliellidae and *Nuttalliella*) for this family and genus were given as an honor to George Nuttall, the bacteriologist who was a notable specialist in tick-borne diseases (Bedford, 1931). The species name is however given based on the collection site (Kamieskroon, in Little Namaqualand) of the first specimen described as *N*. *namaqua* (Bedford, 1931). The above-mentioned author (Bedford, 1931) also hypothesized that this species is a significant link that has been missing in the evolutionary chain of the families Argasidae and Ixodidae. In addition, it is also shown to be closely related to the genus *Ixodes* (Bedford, 1931) as well as the fossil family Deinocrotonidae (Peñalver et al., 2017), however, its life cycle is still not certainly documented. Interestingly, despite the first specimen of *N. namaqua* tick species being described over 90 years ago; its life cycle and the natural hosts of all the life stages are still not certainly defined. Thus, the focus of this review was to answer the following proposed questions (i) What is the distribution of the genus *Nuttalliella*? (ii) Is the species collected from all those countries similar or is there any variation between them? (iii) Is it occurring in minimal or abundant but under documented? (iv) What are its natural preferred hosts? (v) What is the relationship between Nuttalliellidae and other families?

## 2 Materials and methods

In reference to the Preferred Reporting Items for Systematic Reviews and Meta-analyses guidelines for scoping reviews (PRISMA-ScR) (Tricco et al., 2018), a search of literature was conducted by both authors (LMB; DPM) independently in Google Scholar and PubMed databases. Boolean operators were used to combines the search terms and the search criteria for both the databases included the key search terms such as: *Nuttalliella* [AND] occurrence; *Nuttalliella namaqua* [AND] distribution; *Nuttalliella namaqua* [AND] hosts; *Nuttalliella namaqua* [AND] life cycle; Nuttalliellidae [AND] Argasidae [AND] Ixodidae; *Nuttalliella namaqua* [OR] *Nuttalliella species* [AND] Africa (Algeria [OR] Angola [OR] Benin [OR] Botswana [OR] Burkina Faso [OR] Burundi [OR] Cameroon [OR] Cape Verde [OR] Central African Republic [OR] Chad [OR] Comoros [OR] Congo [OR] Côte d’lvoire [OR] Djibouti [OR] DR Congo [OR] Egypt [OR] Equatorial Guinea [OR] Eritrea [OR] Ethiopia [OR] Gabon [OR] Gambia [OR] Ghana [OR] Guinea [OR] Guinea-Bissau [OR] Kenya [OR] Lesotho [OR] Liberia [OR] Libya [OR] Madagascar [OR] Malawi [OR] Mali [OR] Maurinatia [OR] Mauritius [OR] Morocco [OR] Mozambique [OR] Namibia [OR] Niger [OR] Nigeria [OR] Réunion [OR] Rwanda [OR] Sao Tome and Principe [OR] Senegal [OR] Seychelles [OR] Sierra Leone [OR] Somalia [OR] South Africa [OR] South Sudan [OR] Sudan [OR] Swaziland [OR] Tanzania [OR] Togo [OR] Tunisia [OR] Uganda [OR] Western Sahara [OR] Zambia [OR] Zimbabwe). Additional literature searches were done by assessing the references of the initial search output and all the results were filtered and screened by respectively removing the duplicates and reviewing the titles and abstracts. Full text of the articles deemed relevant were downloaded and assessed for eligibility by both authors (LMB; DPM).

The eligibility criteria used in this review, more especially the long range of publication time-limit (1920 to January 2024), was considered because definite insights on the family Nuttalliellidae are still not clearly established; despite the initial species of this family being described more than 90 years ago. Thus, eligibility of the downloaded full text was assessed following the established inclusion criteria which briefly include: (a) Articles published in peer-reviewed journals reporting on the distribution of *N. namaqua* as well as the occurrences and descriptions of *Nuttalliella* species; (b) Articles published from 1920 to at present; (c) Articles that clearly state the tests used in the study; as well as (d) Articles with sample information clearly indicated and described. References that were excluded in the review include: (a) Theses and books; (b) Articles that only mention but do not report on the occurrence, description or distribution of *Nuttalliella*; (c) All types of review articles; (d) Studies that do not contribute towards answering the research questions.

Data was extracted from the included articles and the following information was recorded and used for further appraisal: article authors, sex and life-cycle stage of the collected *N. namaqua* ticks, host or source, geographical localities, diagnostic tests used for screening, outcomes of the included studies. Data was appraised and analyzed using Excel 2016.

## 3 Results

### 3.1 Database search outputs

**Figure 1:**
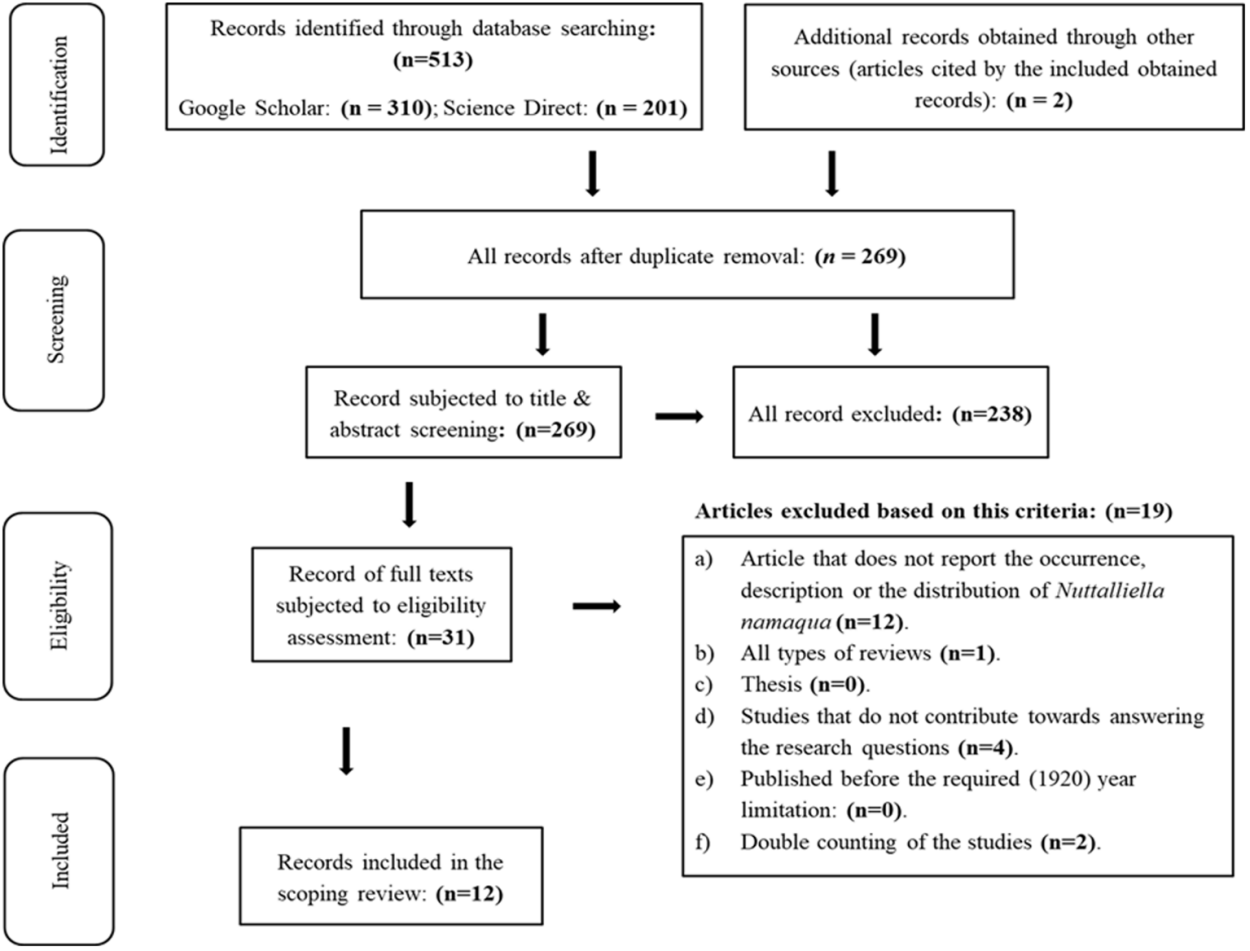
PRISMA flow diagram indicating the search and screening processes.

The databases search as well as the assessment of the articles referenced by the obtained search outputs resulted in a total of 513 references (duplicate articles, all types of reviews, books, thesis and peer-reviewed publications),which were then subjected to filtering by respectively removing the duplicates and screening of the titles and abstracts for relevance (**Figure 1**). A total of 269 records were left after duplicate removal, while 238 were found irrelevant when screening the titles and abstract. Assessment of the downloaded full texts of the remaining 31 articles resulted in the exclusion of 19 more articles and only 12 records were deemed eligible to be included in the review.

### 3.2 Characteristics of the included and appraised articles

**Figure 2:**
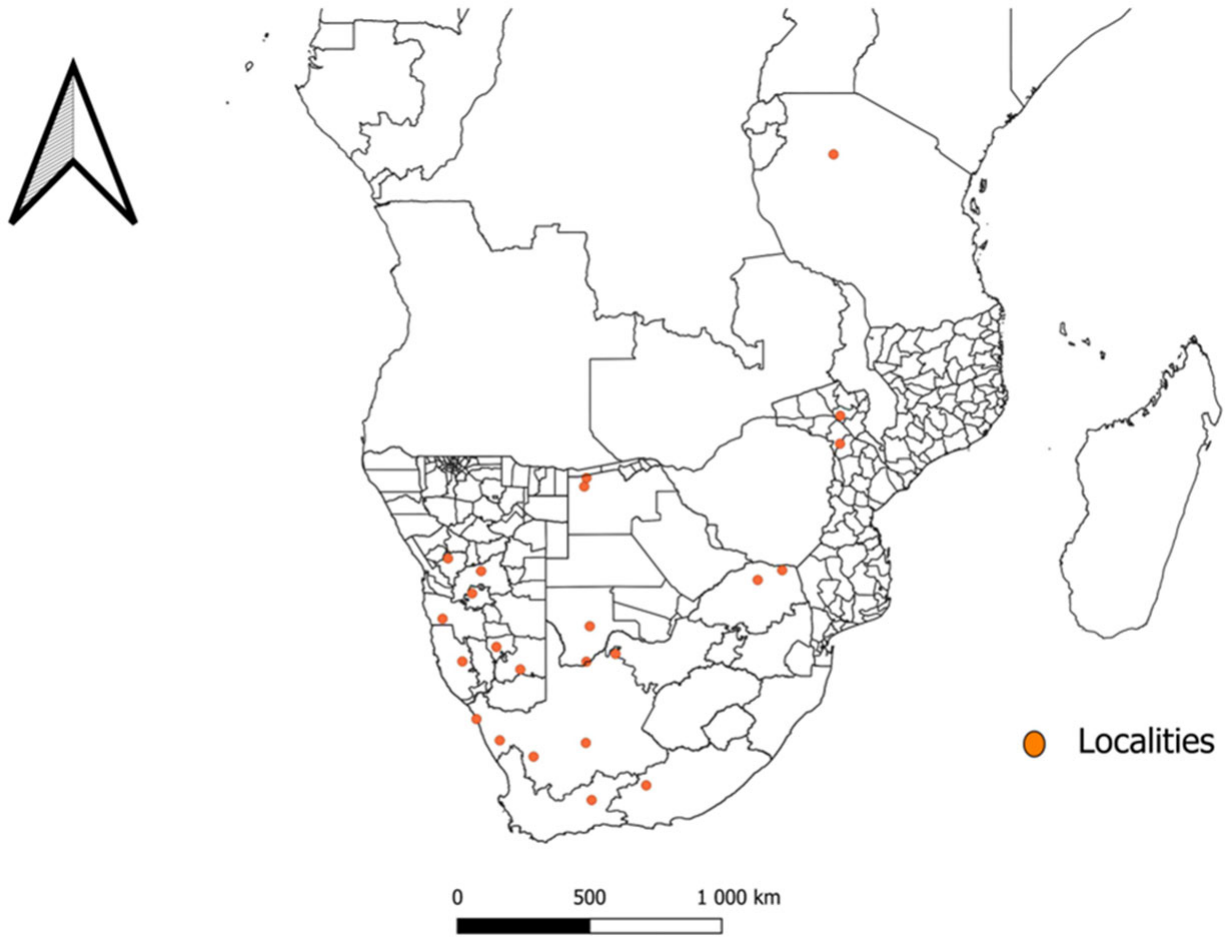
A map indicating the distribution and previous collection site of *Nuttalliella namaqua* in Botswana (Apanaskevich, 2021), Mozambique (Apanaskevich, 2021), Namibia (Keirans et al., 1976; Apanaskevich, 2021), South Africa (Bedford, 1931; Keirans et al., 1976; Mans et al., 2011; Horak et al., 2012; Latif et al., 2012; Apanaskevich, 2021; Stevens et al., 2022) and Tanzania (Stevens et al., 2022).

Data extracted from the included articles (**Table 1**) shows that *N*. *namaqua* was only collected in limited localities in the southern and eastern regions within the African continent. Its previous distribution ranged from South Africa, Tanzania and Namibia, but has recently been recorded for the first time in Botswana and Mozambique as well as new localities in South Africa and Namibia (**Figure 2**). In South Africa, its distribution includes the Namaqualand in the Eastern Cape Province, where it was collected for the first time (Bedford, 1931), Plaatfontein Farm (Horak et al., 2012) Graaff-Reinet (Mans et al., 2011; Latif et al., 2012), Northern Cape Province; Loeriesfontein (Stevens et al., 2022) Gannavloer (Apanaskevich, 2021) Heuningvleipan, North-West Province (Mans et al., 2011; Latif et al., 2012) and the Soutpansberg mountain range (Horak et al., 2012) and Tshipise (Apanaskevich, 2021) in the Limpopo Province. Its distribution in Tanzania includes the Shinyanga District, while in Namibia the species was respectively collected from the Rehoboth and Windhoek (Keirans et al., 1976), as well as Hardap and Karas (Apanaskevich, 2021) districts. Moreover, distribution in Botswana and Mozambique includes North-West and Tete as well as Chiúta and Changara districts respectively (Apanaskevich, 2021).

**Table 1:** Characteristics of the included studies reporting on the description and occurrence of *Nuttalliella namaqua* in African countries as well as the species host preference and life cycle.

Authors	Collected *Nuttalliella namaqua* ticks (life stage and sex)	Host or Source	Localities	Tests used	Results/outcomes
**Bedford, 1931**	Adult (female)	Under a stone	South Africa	Morphology	Description of a new genus and a new species
**Keirans et al., 1976**	Adults (female)	Meerkat (*Suricata suricatta hahni*), Nest of a Lesser Striped Swallow (*Hirundo abyssinica*), rodent (*Parotomys B. brantsi*), under stone	Namibia, South Africa, Tanzania	Morphology	Discovery of *Nuttalliella namaqua* in Tanzania
**Roshdy et al., 1983**	Adult (preserved female)	Not indicated	South Africa	Morphology	Spiracles of *Nuttalliella namaqua* have fenestrated plates that are unique to the family as compared to the sister families Argasidae and Ixodidae
**El Shoura et al., 1984**	Adult (Female)	On the ground	South Africa	Morphology	Nuttalliellidae have internal and external structures similar to other families even though it has its unique features as well
**Mans et al., 2011**	Larvae, nymphs, females and male	Rock crevices (habitats of hyraxes (*Procavia capensis*), lizards and elephant shrews) on a rock wall as well as the collapsed nest of an eagle	South Africa	Molecular	Phylogenetic analysis placed *Nuttalliella namaqua* on the basal position on the tree
**Latif et al., 2012**	As indicated in Mans et al. (2011)	Rock crevices (habitats of hyraxes (*Procavia capensis*), lizards and elephant shrews) on a rock wall as well as the collapsed nest of an eagle	South Africa	Morphology	Redescription of the female and the discovery of the larva, nymphs and the male
**Mans et al., 2012**	As indicated in Mans et al. (2011)	As indicated in Mans et al. (2011)	South Africa	Molecular	Generated the first mitochondrial sequences of Nuttalliellidae and the Argasidae families. Once more, *Nuttalliella namaqua* placed at the basal position on the phylogenetic tree
**Horak et al., 2012**	Larvae	Murid rodents (*Micaelamys namaquensis*, *Aethomys chrysophilus* and *Acomys spinosissimus*)	South Africa	Morphology & molecular	Description of *Nuttalliella namaqua* larva and the report of murid rodents as their natural host. Also shown variation in the 18S rRNA gene of specimen from different localities
**Mans et al., 2014**	Nymphs & females	As indicated in Mans et al. (2011)	South Africa	Molecular	16S analysis of the gut meal revealed that the collected *Nuttalliella namaqua* ticks has fed on several species of lizards as well as the gecko and skinks
**Mans et al., 2015**	As indicated in Mans et al. (2011)	As indicated in Mans et al. (2011)	South Africa	Molecular	Confirmed that Nuttalliellidae is a monotypic family and has a basal correlation with Argasidae and Ixodidae
**Apanaskevich, 2021**	Larvae	African savanna hare (*Lepus microtis*), Black-backed jackal (*Canis mesomela*s), elephant shrew (*Elephantulus rupestris*), Hairy-footed gerbil (*Gerbillurus paeba*), murid rodents (Rodentia: Muridae), scrub hare (*Lepus saxatilis*)	Botswana, Mozambique, Namibia, South Africa	Morphology	Collection of *Nuttalliella namaqua* and records of new localities and new hosts
**Stevens et al., 2022**	Larva	Rodent	South Africa	Morphology	Collection of *Nuttalliella namaqua* from a new locality

^(a)^Only nymphs and females targeted. ^(b)^DNA from only one sample sequenced.

The current review shows that females (Bedford, 1931; Keirans et al., 1976; Roshdy et al., 1983; El Shoura et al., 1984) and nymphs (Latif et al., 2012) of *N. namaqua* were the initial life stages to be described, while larvae (Horak et al., 2012; Latif et al., 2012; Apanaskevich, 2021; Stevens et al., 2022) and males (Latif et al., 2012) were subsequently collected and reported (**Tables 1**, **2**). The included studies also showed that the species was only collected from small mammals (murid rodents, African Savanna hare, scrub hare, elephant shrews, rock hyraxes), black backed jackal, reptiles (various species of lizards), as well as off the host which include under a stone, rock crevices, on a rock wall and respectively in the nests of an eagle and a Lesser Striped Swallow (**Tables 1**, **2**).

**Table 2:** List of the hosts and off-host sources where *N. namaqua* was collected in all the countries in which it occurs.

Localities	Hosts	Life stages (Number of specimens)
**Botswana (** **Apanaskevich, 2021**)	African savanna hare (*Lepus microtis*)	Larva (1)
	Namaqua rock rat (*Micaelamys namaquensis*)	Larva (4)
	Black-backed jackal (*Canis mesomelas*)	Larva (1)
	Red rock rat (*Aethomys chrysophilus*)	Larva (1)
**Mozambique (** **Apanaskevich, 2021**)	*Aethomys* species	Larva (57)
	Red rock rat (*Aethomys chrysophilus*)	Larva (6)
	Southern African spiny mouse (*Acomys spinosissimus*)	Larva (1)
**Namibia (** **Keirans et al., 1976** **;** **Apanaskevich, 2021** **) ^a^**	Four-striped mice (*Rhabdomys pumilio*)	Larva (1)
	Western rock elephant shrew (*Elephantulus rupestris*)	Larva (1)
	Namaqua rock rat (*Micaelamys namaquensis*)	Larva (32)
	Dassie rat (*Petromus typicus*)	Larva (2)
	Hairy-footed gerbil (*Gerbillurus paeba*)	Larva (1)
	Scrub hare (*Lepus saxatilis*)	Larva (1)
	Meerkats (*Suricata suricata*)	Adult (10 females)
**South Africa (** **Bedford, 1931** **;** **Keirans et al., 1976** **;** **El Shoura et al., 1984** **;** **Mans et al., 2011** **;** **Horak et al., 2012** **;** **Apanaskevich, 2021** **;** **Stevens et al., 2022**)	Brants’ whistling rat (*Parotomys brantsii*) **^b^**	Adult (1 female)
	Namaqua rock rat (*Micaelamys namaquensis*)	Larva (155) **^c^**
	Red rock rat (*Aethomys chrysophilus*)	Larva (59) **^d^**
	Littledale’s whistling rat (*Parotomys littledalei*) **^e^**	Larva (4)
	Southern African spiny mouse (*Acomys spinosissimus*)**^f^**	Larva (9)
	Under stone **^g^**	Adult (1 female)
	Rock crevices **^h^**	Adults (14), nymphs (4) & larvae (12)
	Rock wall **^h^**	Adults (female)
	Collapsed eagle nest **^h^**	Nymph (1)
	On the ground **^i^**	Adults (female)
**Tanzania (** **Keirans et al., 1976**)	Nest of the lesser striped swallow	Adults (2 females)

**^a^** All reported by Apanaskevich (2021) except meerkat (Keirans et al., 1976); ^b^reported by Keirans et al. (1976); ^c^154 collected by Horak et al. (2012) while one is collected by Stevens et al. (2022); ^d^58 collected by Horak et al. (2012) while one is collected by Apanaskevich (2021); ^e^reported by Apanaskevich (2021); ^f^reported by Horak et al. (2012); ^g^reported by Bedford (1931); ^h^ reported by Mans et al. (2011); ^i^ reported by El Shoura et al. (1984).

It is also shown that morphological tests were used until a study of Mans et al. (2011), which reported on the phylogenetic analysis of Nuttalliellidae in comparison with Argasidae and Ixodidae (**Table 1**). Only a few articles (n=5/12) (Mans et al., 2011; Horak et al., 2012; Mans et al., 2012, 2014, 2015) used molecular assays. Moreover, the reporting periods of the included studies indicates that it took more than 40 years to collect and report more species (Keirans et al., 1976) after the description of the genus *Nuttalliella* and the initial individual species that was collected under a stone in Kamieskroon, Little Namaqualand (Bedford, 1931).

### 3.3 Life cycle and host preference

Data from this review showed that the initial life stages of *N. namaqua* respectively collected and reported were females (Bedford, 1931; Keirans et al., 1976; Roshdy et al., 1983; El Shoura et al., 1984; Latif et al., 2012) and nymphs (Mans et al., 2011). Larvae and males of this species as well as their possible hosts have ultimately been described after a long time from the initial description of this species (Latif et al., 2012). The review also shows that larvae are currently the most collected specimens of *N. Namaqua*, despite them being described 80 years after the initial description of this species (**Tables 1**, **2**). Only two specimens of males have been collected to date (Latif et al., 2012).

Included articles indicated that this species is a generalist, and its preferred hosts include murid rodents, meerkats (*Suricata suricatta*), different lizards, rock hyraxes (*Procavia capensis*), Brants’ karoo rat (*Parotomys brantsi*), Namaqua rock mouse (*Micaelamys namaquensis*) (**Table 2**). Moreover, murid rodents were shown to be the natural hosts of the *N. namaqua* larvae and have been reported in different localities such as Limpopo and Northern Cape Provinces in South Africa (Horak et al., 2012; Latif et al., 2012; Apanaskevich, 2021; Stevens et al., 2022) as well as Botswana, Mozambique and Namibia (Apanaskevich, 2021). The results also shows that females of this tick were collected from meerkats and the Brants’ whistling rat as well as off the host from collection sites that include under the stone, in rock crevices, on the ground and in the nest of the lesser striped swallow (**Table 2**). Males and nymphs were only collected off the host and were respectively reported on a few occasions (Mans et al., 2011; Latif et al., 2012; Mans et al., 2014).

## 4 Discussions

The current review has shown that *N. namaqua* has a distribution that is limited to African regions that include Botswana (Apanaskevich, 2021), Namibia (Keirans et al., 1976; Apanaskevich, 2021), Mozambique (Apanaskevich, 2021), South Africa (Bedford, 1931; Keirans et al., 1976; Horak et al., 2012; Latif et al., 2012; Stevens et al., 2022) and Tanzania (Keirans et al., 1976). Ticks are distributed globally; however, the distribution of specific tick species varies based on the biotic and abiotic factors such as presence of hosts, vegetation types, altitude, rainfall, humidity and temperature (Jongejan and Uilenberg, 1994). In the southern African region, the distribution range of *N. namaqua* appears to be in arid and semi-arid localities except in Tanzania. It is presumed that the fact that *N. namaqua* species are mainly collected and confined in limited southern African localities, might be an indication that Ixodidae may have originated in Africa (Mans et al., 2011, 2012). In addition, the species shows preference to habitats such as rocky areas, mixed trees and shrub savannah biome (Mans et al., 2011).

The recent description of new *Nuttalliella* species from the fossil specimens collected from the Burmese amber deposits shows that it is probable that the distribution of this genus might also include the Antarctica and Australia (Chitimia-Dobler et al., 2024). This corresponds with the theories of Barker et al. (2014), who speculated that the family Nutalliellidae may have occurred in Australia before the extinction of their preferred hosts, and/or some extant species might still be there but not discovered yet. However, we hypothesize that it might not be the case with the distribution of the extant *N. namaqua* since it was not among the newly described fossil *Nuttalliella* species that has been collected outside the known African regions. On the other hand, further investigations and discovery of this species might provide more insight on its distribution like in the case of *Ixodes scapularis*, which was known to have a distribution limited to the south-eastern United States until it was established that it is a vector of *Borrelia burgdorferi*, the causal agent of Lyme disease in humans (Eisen and Eisen, 2023). Additionally, only 12 articles were included and appraised in the review and the low number of the articles included and appraised in this review might be due to the fact that the species *N. namaqua* is scarce; thus, resulting in low collection rate and/or it is under-investigated or under-reported. This is also shown by the period between the first collection and description of this species (Bedford, 1931) and its second report (Keirans et al., 1976).

The life cycle of all tick species involves four development phases such as the egg, larval, nymphal and adult stages, which varies based on the genus and/or species (Saleh et al., 2021). Despite *Nuttalliella* being an ancient genus, its life cycle is still not clear; and it took more than 80 years before the first specimen of larva and males were collected and described. Thus, due to the abovementioned fact, there were speculation that *N. namaqua* species are parthenogenic, or the males are reserved, or the gender ration of this species is inordinate (Oliver, 1989; Latif et al., 2012). Males were described from the two pairs of ticks that were found in a mating position at different times from Springbok, Namaqualand in South Africa and were later confirmed to be male and female respectively (Latif et al., 2012).

Host preference is generally different among ticks, and it is crucial for tick survival as the extinction of a preferred host can result in the dying out of ticks that cannot be accustomed to the available host (Koh et al., 2004). In case of *N. namaqua*, the current review has shown that the host preference of other life stages except the larva (Horak et al., 2012; Apanaskevich, 2021; Stevens et al., 2022), has not been clearly described. Numerous studies (Keirans et al., 1976; El Shoura et al., 1984; Oliver, 1989) have also shown that the preferred natural host of this species is still uncertain. However, Mans et al. (2012) reported that *N. namaqua* appears to be a generalist since it has been collected from various mammalian hosts, and further investigation of the gut meal from a field collected nymphs and females indicated that they have fed from different lizard species. The above authors (Mans et al., 2012) also speculated that the species has the ability to switch hosts numerously in case of possible extinctions of the preferred hosts. In another study, Horak et al. (2012) has reported that murid rodents are natural hosts of *N. namaqua* larva. Moreover, the authors (Horak et al., 2012) also showed that none of the elephant shrews and hedgehogs found in the same area and examined together with the rodents was infested. Conforming with Horak et al. (2012), a recent study (Apanaskevich, 2021) also collected a large number of larvae from murid rodents and morphologically identified them as *N. namaqua*. Moreover, murid rodents have also been reported as the preferred host of immature stages of several ixodid ticks and ectoparasites in general (Petney et al., 2004; Matthee et al., 2010; Stevens et al., 2022).

Still, the natural hosts parasitized by nymphs of this species are still not known. El Shoura (1991) reported an unsuccessful feeding of nymphs and females on mice, rats, pigeons, chickens and rabbits attempted in the laboratory to further verify the host preference of *N. namaqua*. While Mans et al. (2011) reported their (nymphs and females) successful feeding on lizard in a laboratory setup as well. Numerous specimens were collected off the hosts in localities such as abundant bird nests, on the ground, under the rocks or in the rock crevices (Mans et al., 2011, 2012). This might be because *N. namaqua* can survive for a longer period off-host and without feeding as it can store hemoglobin and red blood cells longer owing to its rapid feeding as well as the slow digestion behavior (Latif et al., 2012). Hence, it has been collected off-host at secluded collection sites that include under a stone, nests of birds as well as the rock crevice, which are habitats of their possible hosts. Thus, allowing the ticks to concurrently quest and be protected from poor climatic conditions.

## 5 Current insights on Nuttalliellidae in relation to other tick families

### 5.1 Morphological analysis

Morphological tests have been used to identify *N. namaqua* in the past, and we speculate that the use of this approach alone was because no DNA was available since there was only one specimen and it was preserved for further investigations and comparison with future collected specimen of this species. Most included studies indicated that the species (all life stages) possesses characteristics that are respectively distinct to hard and soft ticks, and it is presumed to be the living fossil closest to the ancestral tick lineage as well as the missing link between the Argasidae and Ixodidae families (Bedford, 1931; Keirans et al., 1976; El Shoura et al., 1984; Latif et al., 2012). The dorsal pseudoscutum in adults and nymphs of this species are like that of hard ticks, but have a wrinkled cuticle with elevations and pits, a feature distinct to soft ticks (Bedford, 1931; Latif et al., 2012). The initial report of the larva and male specimens of *N*. *namaqua* (Latif et al., 2012) indicated that the larvae have a true scutum, dentate anal plate as well as visible pores on the legs, which are not found in other life stage of this species. It was also indicated that the pores on the legs and the dentate anal plate are also not found in Argasidae and Ixodidae. In addition, the pseudoscutum of *N. namaqua* in males covers most of their dorsal side like that of hard ticks whereas the true scutum of the larvae resemble that of hard tick’s larvae (Latif et al., 2012).

Nonetheless, it also possesses its own unique features, which include the leg segments that are joined by the ball as well as socket-joints that are exceptionally noticeable in adults and nymphs (Latif et al., 2012). In a previous study (Roshdy et al., 1983), it was reported that the spiracles of this species display features of the other two families but also possesses a unique fenestrated plate surface. The larvae of this species exhibit a prolonged feeding behavior while the rapid feeding has been observed in nymphs and adults (Mans et al., 2016). Much like the soft ticks, *N. namaqua* uses its gut as a storage organ for the undigested red blood cells and hemoglobin (Sonenshine and Roe, 2014; Mans et al., 2016). Besides that, it was also reported that the features of *N. namaqua*, specifically the pre-anal groove and the pseudoscutum generally indicates that the species is more related to the genus *Ixodes* in the family Ixodidae than any other genus or family (Bedford, 1931). The family Deinocrotonidae is reported to also possess the ixodid-like pseudoscutum and hypostome features that are similar to those of Nuttalliellidae (Peñalver et al., 2017; Chitimia-Dobler et al., 2024); thus, it is reported that these similarities warrant it to be placed within Nuttalliellidae than to be classified as a family on its own (Chitimia-Dobler et al., 2024). While the newly described Khimairidae differs significantly with both Deinocrotonidae and Nuttalliellidae (Chitimia-Dobler et al., 2022). Khimairidae is reported to possess the features that are a clear combination of hard and soft ticks, making it a better option to be regarded as the last ancestral lineage to Argasidae and Ixodidae families as compared to Deinocrotonidae and Nuttalliellidae (Chitimia-Dobler et al., 2022).

### 5.2 Molecular and systematic analysis of Nuttalliellidae

Molecular approaches have contributed greatly to the phylogeny and systematic studies of ticks and tick-borne diseases. Although they also have some setbacks, their efficacy allows characterization of species to the genus and/or species level targeting varying conserved regions. Ticks are classified in families that have common as well as unique morphological and anatomical features that are influenced by their host and locality (Sonenshine, 1991). The hypotheses concerning the ancestral tick lineages is suspected to be affected by the unresolved phylogenetic position of the family Nuttalliellidae in relation to other tick families (Barker and Murrell, 2002, 2004). This might be due to the fact that a few specimens of *N. namaqua* were available and the efforts to obtain viable DNA of this species was unsuccessful, thus, resulting in the amplification of the DNA from the contaminant fungi in the sample (Barker and Murrell, 2002, 2004). Subsequently, numerous studies (Horak et al., 2012; Mans et al., 2012; Burger et al., 2013, 2014; Mans et al., 2014, 2015) employing markers targeting the nuclear ribosomal RNA and mitochondrial protein-coding genes to further investigate and contribute to the phylogeny of tick families have been carried out. Nuttalliellidae was implicated to be a significant link that has been missing in the evolutionary chain of the families Argasidae and Ixodidae (Bedford, 1931; Latif et al., 2012).

In the contrary, numerous morphological phylogeny studies (Bedford, 1931; Hoogstraal, 1985; El Shoura, 1991) have previously grouped Nuttalliellidae in various positions, thus, resulting in inconsistence assumptions. Pugh (1997) indicated that the Order Ixodida is monophyletic, while Nuttalliellidae is closely related to Argasidae than Ixodidae. Whereas a study targeting the 12S gene (Burger et al., 2014) showed that *Nuttalliella* cluster between the Holothyrida and the Mesostigamata. However, recent studies (Mans et al., 2012, 2014) showed that the three extant tick families phylogenetically group on the same branch, with Nuttalliellidae on the basal spot. In a phylogenetic meta-analysis based on the 18S rDNA sequences of 113 Ixodida taxa, Beati and Klompen (2019), in agreement with the abovementioned authors, reported that *N. namaqua* appeared to be a sister-lineage to all other ticks included in the study.

## 6 Conclusions

Even though it is almost a century since the initial description of Nuttalliellidae, this review shows that its lifecycle is still not completely established, and the distribution of the extant *N. namaqua* is still limited to a few African countries. Most adult ticks were collected in South Africa, with only a few from Namibia and Tanzania. Even though it combines some features of both the Argasidae and Ixodidae, it also possesses its unique characteristics. *Nuttalliella namaqua* is also not linked to any disease-transmitting pathogen since its description. Limited knowledge of the family Nuttalliellidae as well as the recently described fossil families Deinocrotonidae, Khimairidae and Legionaris extend the gap and hinder the advances in determining the tick phylogeny and ancestral lineage.

## Data availability statement

The original contributions presented in the study are included in the article/supplementary material, further inquiries can be directed to the corresponding author/s.

## Author contributions

ML: Writing – original draft, Writing – review & editing, Conceptualization, Data curation, Formal analysis, Methodology, Project administration, Validation. DM: Writing – original draft, Writing – review & editing, Conceptualization, Data curation, Methodology, Project administration, Resources, Supervision, Validation.
